# Lovastatin-Induced Mitochondrial Oxidative Stress Leads to the Release of mtDNA to Promote Apoptosis by Activating cGAS-STING Pathway in Human Colorectal Cancer Cells

**DOI:** 10.3390/antiox13060679

**Published:** 2024-05-31

**Authors:** Xiaoming Huang, Ning Liang, Fuming Zhang, Wanjun Lin, Wenzhe Ma

**Affiliations:** 1 State Key Laboratory of Quality Research in Chinese Medicine, Macau University of Science and Technology, Macau 999078, China; 2 Institute of Biopharmaceutical and Health Engineering, Tsinghua Shenzhen International Graduate School, Tsinghua University, Shenzhen 518055, China; 3 Tsinghua Berkeley Shenzhen Institute, Tsinghua Shenzhen International Graduate School, Tsinghua University, Shenzhen 518055, China; 4 College of Physics and Optoelectronic Engineering, Shenzhen University, Shenzhen 518060, China; 5 School of Pharmacy, Shenzhen University Medical School, Shenzhen University, Shenzhen 518055, China

**Keywords:** human colorectal cancer, lovastatin, ROS, DNA damage, cGAS-STING, apoptosis

## Abstract

Statins are 3-hydroxy-3-methylglutaryl coenzyme-A (HMG-CoA) reductase inhibitors widely used in the treatment of hyperlipidemia. The inhibition of HMG-CoA reductase in the mevalonate pathway leads to the suppression of cell proliferation and induction of apoptosis. The cyclic GMP-AMP synthase (cGAS) stimulator of the interferon genes (STING) signaling pathway has been suggested to not only facilitate inflammatory responses and the production of type I interferons (IFN), but also activate other cellular processes, such as apoptosis. It has not been studied, however, whether cGAS-STING activation is involved in the apoptosis induced by statin treatment in human colorectal cancer cells. In this study, we reported that lovastatin impaired mitochondrial function, including the depolarization of mitochondrial membrane potential, reduction of oxygen consumption, mitochondrial DNA (mtDNA) integrity, and mtDNA abundance in human colorectal cancer HCT116 cells. The mitochondrial dysfunction markedly induced ROS production in mitochondria, whereas the defect in mitochondria respiration or depletion of mitochondria eliminated reactive oxygen species (ROS) production. The ROS-induced oxidative DNA damage by lovastatin treatment was attenuated by mitochondrial-targeted antioxidant mitoquinone (mitoQ). Upon DNA damage, mtDNA was released into the cytosol and bound to DNA sensor cGAS, thus activating the cGAS-STING signaling pathway to trigger a type I interferon response. This effect was not activated by nuclear DNA (nuDNA) or mitochondrial RNA, as the depletion of mitochondria compromised this effect, but not the knockdown of retinoic acid-inducible gene-1/melanoma differentiation-associated protein 5 (RIG-I/MDA5) adaptor or mitochondrial antiviral signaling protein (MAVS). Moreover, lovastatin-induced apoptosis was partly dependent on the cGAS-STING signaling pathway in HCT116 cells as the knockdown of cGAS or STING expression rescued cell viability and mitigated apoptosis. Similarly, the knockdown of cGAS or STING also attenuated the antitumor effect of lovastatin in the HCT116 xenograft model in vivo. Our findings suggest that lovastatin-induced apoptosis is at least partly mediated through the cGAS-STING signaling pathway by triggering mtDNA accumulation in the cytosol in human colorectal cancer HCT116 cells.

## 1. Introduction

Statins, inhibitors of 3-hydroxy-3-methylglutaryl coenzyme A (HMG-CoA) reductase, a rate-limiting enzyme of the mevalonate pathway, are widely used as lipid-lowering drugs to reduce cardiovascular events and death [1]. However, in the past decades, independent studies suggested that statins display pleiotropic effects independent of their lipid-lowering capabilities, including but not limited to inhibiting tumor growth and inducing apoptosis in specific cancer cell types [1,2]. Dysregulation of the mevalonate pathway has been shown in different cancers including melanoma, breast, lung, and colorectal cancer, etc. [3]. Notably, all-cause mortality (ACM) and cancer-specific mortality (CSM) among patients with cancer who are taking statins are both reduced by 15% [4]. A meta-analysis demonstrates that the use of statins is not only associated with a 15% reduced risk of ACM and 18% reduced risk of CSM in pre-diagnosis for CRC patients but also associated with a 14% reduced risk of ACM and 21% reduced risk of CSM in post-diagnosis for CRC patients [5]. Therefore, statins have a pivotal role in cancer prevention as well as treatment. However, the mechanisms underlying the anticancer effects of statin in this context are not yet fully established.

Mitochondria are intracellular membrane bound organelles present in almost all eukaryotic cells [6]. The most important function of these organelles is to produce energy through the process of oxidative phosphorylation (OXPHOS) [7]. Furthermore, mitochondria are also involved in many other cellular functions, such as the regulation of cell proliferation, differentiation, and death. Their different roles in various cellular processes are largely dependent on reactive oxygen species (ROS) and ATP production, both of which are generated during OXPHOS [7]. Moreover, mitochondria are not only an important source of ROS but also the target of ROS, since excessive ROS production can lead to severe cellular damage and cell death [8]. The oxidative damage in mitochondria can result in a continuous cycle of ROS generation containing superoxide (O_2_^−^) and hydrogen peroxide (H_2_O_2_^−^) [9], which further amplifies oxidative damage to mitochondrial DNA (mtDNA) and proteins, leading to the alteration of cellular function, such as metabolism and survival [10]. In addition to the reduction of cholesterol levels, statins reduce farnesyl pyrophosphate, an intermediate in the synthesis of CoQ10 (a powerful redox substance in the mitochondrial electron transport chain), in the mevalonate pathway [11]. Furthermore, a previous study also demonstrated that statins can directly inhibit mitochondrial complex III and disrupt the mitochondrial membrane potentials [12]. On the one hand, the inhibition of the synthesis of CoQ10 in turn leads to the exacerbation of oxidative stress and cellular damage. On the other hand, the direct inhibition of complex III by statins also can lead to the generation of ROS in mitochondria. Therefore, statins are known to induce mitochondrial dysfunction, which is manifested as myotoxicity, the most adverse effect of statins [13]. Consistently, statin-associated impairment of the function of mitochondria has been reported to increase oxidative stress, not only in human skeletal muscle cells [14] but also in different types of cancer cells [1]. The dysfunction of mitochondria will increase ROS production and cause damage to mtDNA [15,16]. Although our previous research revealed that lovastatin can inhibit ATP production and oxygen consumption (OCR) in colorectal cancer cells (CRCs), the impact of lovastatin on ROS production has not yet been investigated [17]. Therefore, we speculated that lovastatin may increase the production of ROS and result in damage to mtDNA in CRCs.

Sensing of microbial pathogens and tissue damage by the innate immune system triggers host cells to secrete cytokines that promote host defense. Cytosolic DNA, viral RNA, and the bacterial lipopolysaccharide (LPS) activate signaling cascades through a large family of pattern recognition receptors (PRRs), including cGAS/STING, RIG-I/MDA5 (retinoic acid-inducible gene-1, RIG-I; melanoma differentiation-associated protein 5, MDA5), and TLR3/4-TRIF (TLR3/4, Toll-like receptors 3/4) [18]. The recognition of DNA serves as an immune-stimulatory molecule and is an evolutionarily conserved mechanism that rapidly triggers innate immunity against microbial pathogens [19]. The cyclic GMP-AMP (cGAS)-stimulator of the interferon genes (STING) pathway has emerged as critical DNA-sensing machinery involved in innate immunity and viral defense [19]. cGAS recognizes double-strand DNA (dsDNA) to form a complex that initiates conformational conversion, allowing cGAS to catalyze adenosine triphosphate (ATP) and guanosine triphosphate (GTP) into a cyclic dinucleotide, 2′,3′-cyclic GMP-AMP (cGAMP). This subsequently activates STING in the endoplasmic reticulum (ER) [20]. Then, STING is transported to the Golgi apparatus through the ER–Golgi intermediate compartment [21]. After translocating to Golgi, STING is palmitoylated to recruit TANK-bind kinase 1 (TBK1), which in turn phosphorylates STING in the C-terminal and promotes interferon (IFN) regulatory factor 3 (IRF3) and NFκB translocation to the nucleus and upregulation of type I IFNs, proinflammatory cytokines, and chemokines transcription [18,22,23]. The dsDNA bound to cGAS is not limited to exogenous DNA from pathogens such as bacteria and viruses. cGAS can also bind to endogenous DNA including mtDNA, genomic DNA, and DNA in the micronuclei and retrotransposon implicated in cellular stress and damage [24,25,26]. Additionally, RIG-I-like receptors (RLRs) are an important family of pattern recognition receptors which have a critical role in activating the immunological responses against viral RNAs through interaction with mitochondrial antiviral signaling protein (MAVS) in the cytoplasm [27]. Recent studies have demonstrated that not only viral RNAs but also host-derived RNAs can trigger RLR activation, resulting in the production of type I and III IFNs and inflammatory cytokines [28,29].

Recent work has shown that DNA damage-induced apoptosis is dependent on cGAS-STING pathway activation [30,31,32]. Statins have been widely reported to exhibit anticancer activity by inducing apoptosis. However, it has not been investigated whether cGAS-STING activation contributes to statin-induced apoptosis. Thus, we speculate that statin-induced apoptosis may be induced by the activation of the cGAS-STING pathway, which is triggered by the release of mtDNA into the cytosol resulting from mitochondrial oxidative DNA damage.

In this study, we evaluated the impact of lovastatin on mitochondrial dysfunction by investigating mitochondrial ROS production and mtDNA damage in the CRC cell line HCT116. We also investigated the effect of mtDNA release into the cytosol caused by lovastatin on cGAS-STING activation, as well as the relationship between cGAS-STING activation and lovastatin-induced apoptosis in vitro and in vivo. Our study demonstrates that lovastatin-induced oxidative stress promotes the accumulation of mtDNA in the cytosol and activates the cGAS-STING pathway, ultimately leading to apoptosis.

## 2. Materials and Methods

### 2.1. Materials

Lovastatin, carbonyl cyanide phospho-(p)-trifluoromethoxy phenylhydrazone (FCCP), carbonyl cyanide 3-chlorophenylhydrazone (CCCP), oligomycin, rotenone, antimycin A, and uridine were purchased from Sigma Aldrich (St. Louis, MO, USA). Ethidium bromide (EtBr) and sodium pyruvate were obtained from Life Technology (Carlsbad, CA, USA). Mitoquinone (MitoQ) mesylate, H-151, and RU.521 were purchased from TargetMol (Boston, MA, USA).

### 2.2. Cell Culture

Human CRC cell line HCT116 and human embryonic kidney cell line HEK293T were purchased from ATCC (Manassas, VA, USA). HCT116 SCO2−/− cell line was a kind gift from Dr. Paul M. Hwang at the Laboratory of Cardiovascular and Cancer Genetic, National Institute of Health, Bethesda, MD, USA. HCT116, HEK293T, and HCT116 SCO2−/− cells were cultured in Dulbecco’s Modified Eagle’s medium (DMEM; Gibco, NY, USA) containing 10% fetal bovine serum (FBS; Gibco, NY, USA), 100 units/mL penicillin, and 100 mg/mL streptomycin at 37 °C in a humidified atmosphere with 5% CO_2_.

### 2.3. Cell Transfection and Lentivirus for Gene Knockdown

To generate shRNA knockdown lentivirus, shRNAs were cloned into pLKO.1-puro plasmid (Addgene plasmid #8453). shRNA-targeting *MAVS* (TRCN0000236031), *cGAS* (TRCN0000149984), and *STING* (TRCN0000160281) were obtained from the Lenti-shRNA Core Facility (UNC Chapel Hill). HEK293T cells were transfected with 0.5 μg of plasmid of interested genes, 0.5 μg of MISSION packing mix plasmid (Sigma, St. Louis, MO, USA) using FuGENE HD (Promega, Madison, WI, USA), and the lentivirus was collected as previously described [17]. HCT116 cells were transduced with the lentivirus using 8 μg/mL polybrene. After overnight transduction, the transduction medium was changed. Cells were selected with 2 μg/mL puromycin for 2–3 days. Gene knockdown was then confirmed by using immunoblotting. 

### 2.4. Antibodies

Primary antibodies against human proteins, mouse monoclonal anti-phospho-Histone H2A.X (γH2AX; #05-636-1) and mouse monoclonal anti-8-oxoguanine (#MAB3650), were purchased from Millipore (Billerica, MA, USA). Rabbit monoclonal anti-glyceraldehyde-3-phosphate dehydrogenase (GAPDH, #5174), rabbit monoclonal anti-cyclic GMP-AMP synthase (cGAS; #15102), rabbit monoclonal anti-stimulator of interferon genes (STING; #13647), rabbit monoclonal anti-phospho-STING (p-STING; #50907), rabbit monoclonal anti-TANK binding kinase 1 (TBK1; #3504), rabbit monoclonal anti-phospho-TBK1 (p-TBK1; #5483), rabbit monoclonal anti-interferon regulatory factor 3 (IRF3; #11904), rabbit monoclonal anti-phospho-IRF3 (p-IRF3; #29047), rabbit monoclonal anti-phospho-NF-κB p65 (p-NF-κB p65; #8242), rabbit monoclonal anti-NF-κB p65 (NF-κB p65; #3033), mouse monoclonal anti-PARP (#9532), and rabbit polyclonal anti-cleaved caspase-3 (#9661) were from Cell Signaling Technology (Ipswich, MA, USA). Mouse monoclonal anti-Tom20 (#sc17764) was from Santa Cruz Biotechnology (Dallas, TX, USA). Rabbit polyclonal anti-Flag (#ab1162) was from Abcam (Cambridge, MA, USA). Rabbit polyclonal anti-mitochondrial antiviral signaling protein (MAVS; #ab1162) was from Abclonal (Wuhan, China). Anti-rabbit (#211-035-109) and anti-mouse (#715-035-150) secondary antibodies were obtained from Jackson ImmunoResearch Laboratories (West Grove, PA, USA).

### 2.5. Cell Viability Assay

A cell proliferation assay was performed using the sulforhodamine B (SRB) assay, as previously described [33]. Cells (5 × 10^3^ cells/mL) were seeded in 96-well plates at a volume of 100 μL per well and incubated overnight. After incubation, 100 μL of the medium containing indicated drugs was added. Cells were fixed with 50 μL cold 50% (*w*/*v*) trichloroacetic acid (TCA) at 4 °C for 1 h and subsequently stained with 0.4% (*w*/*v*) SRB at room temperature for 30 min. OD_515nm_ was determined using the SpectraMax paradigm microplate reader (Molecular Devices) following the addition of 200 μL of 10 mM tris base solution (pH 10.5). For crystal violet staining, cells were seeded in 12-well plates, the cells were treated with indicated drugs after reaching 20–30% confluency. After treatment, cells were washed, fixed, and stained with 0.4% crystal violets solution.

### 2.6. Mitochondrial DNA-Depleted ρ^0^ Cells

HCT116 cells were cultured in DMEM containing 100 ng/mL ethidium bromide (EtBr), 100 μg/mL pyruvate, and 50 μg/mL uridine as previously described [34]. HCT116 cells were maintained in this medium for three weeks and then cultured in the presence of uridine to achieve mtDNA-depleted cells. The depletion of mtDNA was analyzed using real-time PCR to measure the expression of nuclear *18S rRNA* and the mitochondrial *MTCO2* gene.

### 2.7. Immunoprecipitation/PCR

HCT116 cells transfected with an expression plasmid (pCDNA3.1-FLAG-cGAS; Miaoling Bio, Wuhan, China) encoding Flag-tagged human cGAS were selected with 0.5 mg/mL G418. Then, the cells stably expressing Flag-cGAS were isolated as single clones by limiting dilution. Stable HCT116 cells were then treated with lovastatin with the indicated concentration for 48 h. Following crosslinking of the DNA and associated proteins, immunoprecipitation was carried out with anti-Flag M2 (Sigma, St. Louis, MO, USA) and coprecipitated DNA was determined by real-time qPCR. The method was the same as described in a previous study [35,36]. Oligonucleotide sequences are listed in Table S1.

### 2.8. Quantitative Real-Time PCR

After treatment, total RNA was isolated by binding to poly (dT) magnetic Dynabeads (Life Technologies, Carlsbad, CA, USA) and reverse transcribed to cDNA using SuperScript III (Invitrogen, Carlsbad, CA, USA) according to the manufacturer’s instructions. A quantitative real-time PCR was performed using SYBR Green (Molecular Probes, Eugene, OR, USA) on a ViiA™ 7 Real-Time PCR System (Applied Biosystems, Forster City, CA, USA). The expression values of each sample were quantified and normalized against the eukaryotic translation initiation factor (*EIF3S5*) cDNA using the 2^−ΔΔCT^ method. A mitochondrial DNA copy number analysis was performed as previously described using primers specific to nuclear *18S RNA* and themitochondrial *MTCO2* gene [37]. Oligonucleotide sequences are listed in Table S2. 

### 2.9. mtDNA Determination in Cytosolic Extracts

Cytosolic mtDNA was determined as previously described [38]. Briefly, HCT116 cells were exposed to lovastatin for 48 h, then 1 × 10^7^ cells were aliquoted into two tubes. One aliquot was resuspended in 500 μL of 50 mM and boiled for 30 min to solubilize total DNA, following adding 50 μL of 1 M Tris-HCl (pH 8.0) to neutralize the pH, and these total extracts served as normalized controls for total mtDNA. The second aliquot was resuspended in 500 μL digitonin buffer [150 mM NaCl, 50 mM HEPES (pH 7.4), and 15–25 μg/mL digitonin]. The suspensions were incubated for 10 min with an end over end rotation, then centrifuged at 980× *g* for 3 min three times. The cytosolic supernatant was transferred to a new tube and centrifuged at 17,000× *g* for 10 min. Then, DNA was extracted using QIAQuick Nucleotide Removal Columns (QIAGEN, Hilden, Germany). A real-time PCR was performed to detect the cytosolic mtDNA as relative abundance to *18S RNA* in total extracts, presented as the ratio of cytosolic compared to the overall input of total extracts.

### 2.10. Annexin V/PI Staining

Apoptotic cells were stained with FITC Annexin V and PI and analyzed by flow cytometry as previously described [17]. Briefly, cells were plated in 6-well plates and treated with the indicated drugs. After treatment, the cells were harvested, washed, resuspended in 1 × Binding Buffer, stained with FITC Annexin V and PI, and analyzed by using flow cytometry.

### 2.11. Seahorse XFp Respirometry Assay

The oxygen consumption rate (OCR) of HCT116 cells was measured using an XFp Extracellular Flux Analyzer (Seahorse Bioscience, Billerica, MA, USA). Cells were seeded at 5 × 10^3^ cells/well in XFp cell culture miniplates and treated with lovastatin. After incubation for 48 h, cells were washed and incubated in the XF assay medium at 37 °C in a CO_2_-free incubator for 1 h. The baseline OCR was recorded 3 times with an interval of 6 min. Three subsequent injections followed, comprising 1 μM oligomycin, 1 μM FCCP, and 0.5 μM rotenone/antimycin. The OCR was automatically recorded and calculated by the Seahorse XFp Wave software version 2.6.1.

### 2.12. ROS Measurement

Intracellular ROS levels were measuring using a flow cytometer with CM-H_2_DCFDA stain (Molecular Probes, Eugene, OR, USA) following the manufacturer’s instructions. After treatment, cells were washed in PBS, trypsinized, and resuspended in a complete medium at a concentration of 6 × 10^5^ cells/mL. The resuspended cells were stained with 5 μM CM-H_2_DCFDA for 30 min at 37 °C, washed, and resuspended in PBS. The data were acquired from 10,000 events with a FACSAria II flow cytometer (BD Bioscience, San Jose, CA, USA) and analyzed with FlowJo software version 10.8.1 (TreeStar, Ashland, OR, USA).

### 2.13. Mitochondrial ROS Measurement

Mitochondrial ROS production was measuring using a flow cytometer and MitoSOX Red superoxide indicator (Molecular Probes, Eugene, OR, USA) following the manufacturer’s instructions. For a positive control, cells were pretreated with 20 μM antimycin A for 40 min. After treatment with the indicated drugs for the indicated time periods, cells were washed in PBS, trypsinized, and resuspended in the complete medium at a concentration of 5 × 10^5^ cells/mL. The resuspended cells were stained with 2 μM MitoSOX Red for 30 min at 37 °C, washed, and resuspended in PBS. The data were acquired from 10,000 events with a FACSAria II flow cytometer (BD Bioscience, San Jose, CA, USA) and analyzed with FlowJo software (TreeStar, Ashland, OR, USA). 

### 2.14. Mitochondrial Membrane Potential Measurement

Mitochondrial membrane potential was measuring using a flow cytometer with TMRE stain (Beyotime, Shanghai, China) following the manufacturer’s instructions. After treatment, cells were washed in PBS, trypsinized, and resuspended in a serum-free medium at a concentration of 1 × 10^6^ cells/mL. The resuspended cells were stained with 10 μM TMRE for 20 min in the dark at 37 °C. For the positive control, one sample was treated with 25 μM CCCP for 5 min at 37 °C prior to staining. Then, cells were washed twice in PBS and resuspended in PBS. The data were acquired from 10,000 events with a FACSAria II flow cytometer (BD Bioscience, San Jose, CA, USA) and analyzed with FlowJo software (TreeStar, Ashland, OR, USA).

### 2.15. Immunoblotting

Protein samples were lysed in a RIPA buffer supplemented with a complete protease inhibitor cocktail (Roche, Mannheim, Germany) and phosphatase inhibitor (Roche). Proteins in mitochondrial and cytosolic fractions were isolated using the Qproteome Mitochondria Isolation Kit (QIAGEN, Hilden, Germany). Samples were spun clear of the insoluble fraction and boiled at 95 °C for 5 min in a protein-loading buffer. Samples were separated on sodium dodecyl sulfate-polyacrylamide (SDS-PAGE) gels and transferred onto polyvinylidene difluoride (PVDF) membranes (Millipore). Membranes were blocked in 5% skim milk prepared in 1× tris buffered saline with 0.1% tween 20 (1 × TBST) for 1 h before incubating with primary antibodies at 4 °C overnight. Membranes were washed with 1 × TBST and incubated with horseradish peroxidase (HRP)-conjugated secondary antibodies at room temperature for 1 h. Membranes were visualized using an Amersham Imager 600 (GE Healthcare Life Sciences, Marlborough, MA, USA) with SuperSignal West Dura Extended Duration Substrate or SuperSignal West Pico Chemiluminescent Substrate (Thermo Scientific, San Jose, CA, USA).

### 2.16. Mitochondrial DNA Integrity Determination

Cells were plated at 15 × 10^4^ cells/well in a 6-well plate, incubated overnight, and then treated with drugs for the indicated time. Total DNA was extracted from cells according to the DNeasy Blood & Tissue Kit (QIAGEN, Hilden, Germany). For the mtDNA assay, short (222 bp) and long (8.9 kb) mtDNA fragments reflecting the copy number and mtDNA integrity, respectively, were amplified using the GeneAmp XL PCR Kit (Applied Biosystems, Forster City, CA, USA) and quantified using PicoGreen (Molecular Probes, Eugene, OR, USA) as described previously [39]. The relative mtDNA integrity was calculated as a ratio of the amounts of long versus short PCR fragments.

### 2.17. Immunocytochemistry Staining

Cells were cultured on Cellvis 35 mm glass-bottom dishes (Cellvis, Mountain View, CA, USA) and treated with the indicated drugs for the indicated time. Then, cells were washed twice with ice-cold PBS, fixed with 4% paraformaldehyde, and permeabilized with 0.1% Triton X-100 in PBS. After washing twice with ice-cold PBS, the cells were blocked with 3% bovine serum albumin (BSA) in PBS and incubated with anti-8-oxoguanine (8-oxoG) antibody at 4 °C overnight. Then, the cells were washed twice with ice-cold PBS and subsequently stained with FITC-conjugated secondary antibodies (BD PharMingen, San Diego, CA, USA). Finally, the cell nucleus was visualized by counterstaining DAPI (Roche, Mannheim, Germany). Images were captured using the Olympus IX71 inverted microscope (Olympus, Tokyo, Japan) or Zeiss LSM SP8 laser confocal microscope (Zeiss LSM, Oberkochen, Germany).

### 2.18. Total DNA 8-oxoG Content Measurement

Cells were plated in 6-well plates and treated with indicated drugs for the indicated time. Total DNA was isolated using the DNeasy kit (QIAGEN, Hilden, Germany) and 8-oxoG content determined using an 8-OHdG ELISA kit following manufacturer’s protocol (BioVendor, Brno, Czech Republic). Absorbance signals were collected using the SpectraMax paradigm microplate reader (Molecular Devices, Sunnyvale, CA, USA).

### 2.19. Animal Experiments

Six- to eight-week-old female BALB/c athymic nude mice were subcutaneously injected with 3 × 10^6^ cells suspended in 100 μL of PBS containing 50% growth factor reduced matrigel. When the tumor size reached around 100 mm^3^, the nude mice received one of the following treatments: (i) control (DMSO); (ii) lovastatin (20 mg/kg), intraperitoneally, daily for 2 weeks. The xenograft sizes were measured using a digital caliper, and the volumes were calculated following the formula = (length × width^2^)/2. All animal experiments were carried out following the guidelines of the Division of Animal Control and Inspection (DACI) of the Department of Food and Animal Inspection and Control (DFAIC) of Macau and approved by the Animal Care and Use Committee (ACUC) of the Macau University of Science and Technology (MUST).

### 2.20. Statistical Analyses

Data are typically mean ± SD unless otherwise indicated and were analyzed by an unpaired two-tailed Student’s *t* test between two groups or a one-way analysis of variance (ANOVA) followed by Tukey’s multiple comparison test as appropriate using GraphPad Prism 8.0.2 software (San Diego, CA, USA) (* *p* < 0.05; ** *p* < 0.01; *** *p* < 0.001; ns, no significance). 

## 3. Results

### 3.1. Lovastatin Exposure Induces Mitochondrial Dysfunction in HCT116 Cells

**Figure 1:**
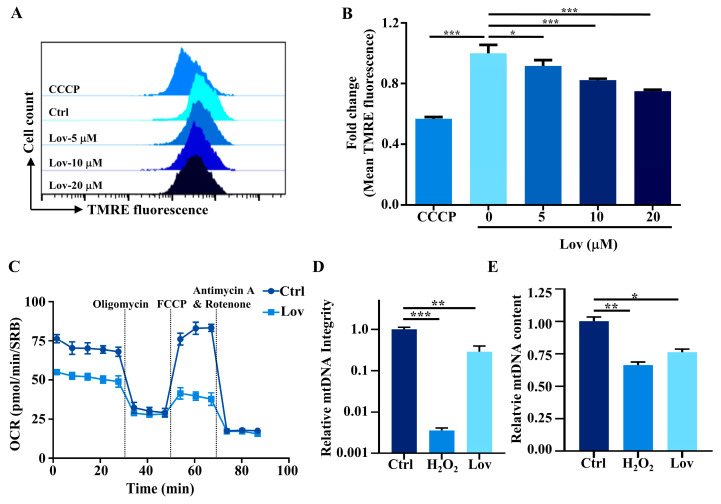
Lovastatin exposure induces mitochondrial dysfunction in HCTT116 cells. (**A**) After treatment with different concentration of lovastatin for 48 h, mitochondrial membrane potential of HCT116 cells was analyzed in the fluorescence intensity of TMRE by flow cytometry. CCCP served as depolarization control. (**B**) Quantification data of TMRE fluorescence intensity of (**A**). (**C**) After treatment with lovastatin (10 μM) for 48 h, oxygen consumption rate (OCR) was measured using a Seahorse XFp analyzer and normalized to protein content determined by using SRB assay. (**D**) HCT116 cells were either treated with lovastatin (10 μM) for 48 h or H_2_O_2_ (100 μM) for 1 h as a positive control, then the relative mitochondrial DNA (mtDNA) integrity was measured by quantitative PCR analysis. (**E**) Quantitative PCR analysis of mtDNA abundance of HCT116 cells treated with lovastatin (10 μM) for 48 h or H_2_O_2_ (200 μM) for 8 h as a positive control. All data were obtained from at least three independent experiments. * *p* < 0.05, ** *p* < 0.01, *** *p* < 0.001.

Membrane potential serves as a functional indicator of mitochondrial integrity. To investigate whether the lovastatin impaired the function of mitochondria, we utilized TMRE staining and flow cytometry to detect the mitochondrial membrane potential (MMP) of HCT116 treated with lovastatin (Figure 1A,B). The findings revealed a dose-dependent depolarization of MMP following lovastatin treatment. The primary function of mitochondria is to produce energy, and the most appropriate way to determine the overall mitochondrial energy production efficiency is to measure the mitochondrial oxygen consumption (OCR) [40]. In line with our previous work [17], lovastatin significantly decreased the OCR in HCT116 cells (Figure 1C). To investigate the effect of lovastatin on the integrity of the mitochondria genome, we examined the mtDNA integrity using a PCR-based assay (quantitative long PCR), which is sensitive in measuring mtDNA breaks [39]. As shown in Figure 1D, the exposure of HCT116 cells to H_2_O_2_ as the positive control significantly reduced mtDNA integrity, in line with a previous study [41]. As expected, the results demonstrated that the mtDNA integrity of the lovastatin treatment group was significantly lower than that of the control group (Figure 1D). Consistent with this finding, a quantitative PCR (qPCR) analysis revealed that lovastatin significantly decreased the abundance of mtDNA (Figure 1E).

### 3.2. Lovastatin Exposure Induces DNA Damage in HCT116 Cells

**Figure 2:**
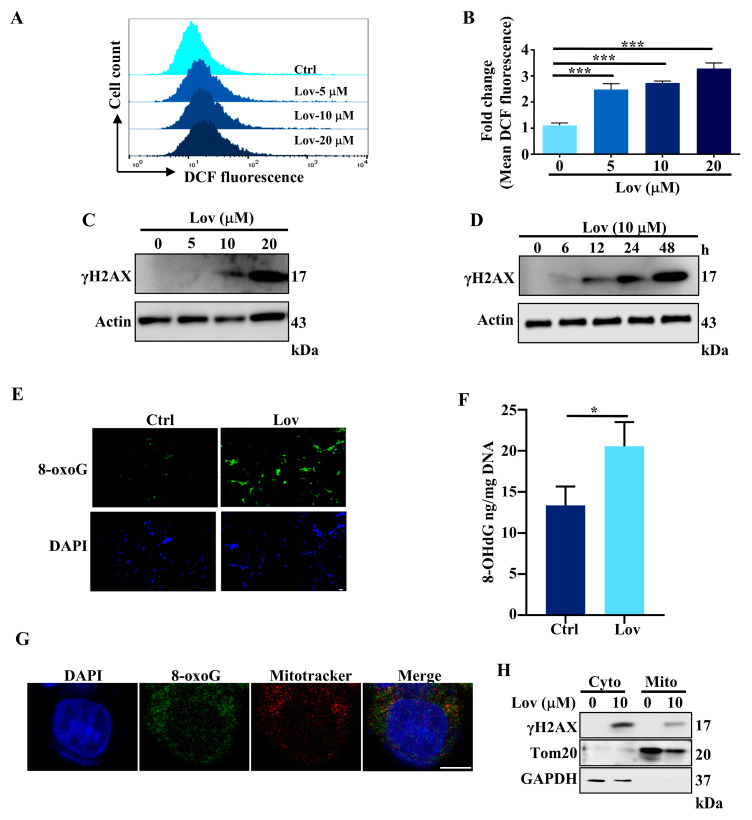
Lovastatin exposure induces DNA damage in HCT116 cells. (**A**) After treatment with a different concentration of lovastatin for 48 h, the ROS production was assessed according to the changes in the fluorescence intensity of DCF by flow cytometry. (**B**) Quantification data of DCF fluorescence intensity of (**A**). (**C**) Representative immunoblots of γH2AX from HCT116 cells treated with different concentrations of lovastatin for 48 h. β-Actin served as a loading control. (**D**) Representative immunoblots of γH2AX from HCT116 cells treated at different time points. (**E**) HCT116 cells were treated with lovastatin (10 μM) for 48 h. Furthermore, 8-oxoG was detected by immunofluorescence staining. Bar, 50 μm. (**F**) The content of 8-OHdG was quantified using 8-OHdG ELISA enzyme-linked immunosorbent assay in HCT116 cells treated with lovastatin (10 μM) for 48 h. (**G**) After treatment with 10 μM lovastatin for 48 h, cells was stained with Lysotracker Red DND99, followed by fixation and permeabilization, then 8-oxoG was detected by immunofluorescence staining. Bar, 5 μm. (**H**) Representative immunoblots of γH2AX, Tom20, and GAPDH in mitochondrial and cytosolic fractions from HCT116 cells treated with 10 μM lovastatin for 48 h. All data were obtained from at least three independent experiments. * *p* < 0.05, *** *p* < 0.01.

Since mitochondrial dysregulation is closely associated with excessive ROS production [42], we treated HCT116 cells with a serial concentration of lovastatin for 48 h to determine its effect on ROS production. The results showed that lovastatin treatment led to an increase in intracellular ROS accumulation (Figure 2A,B). ROS are one of the oxidants which has modulatory effects on DNA damage response, integrating the DNA damage response, DNA repair, and DNA replication [43]. We next determined whether lovastatin induces DNA damage by measuring the level of γH2AX, a sensitive molecular marker of DNA damage and repair response. After the treatment of the HCT116 cells with increasing concentrations of lovastatin for 48 h, the immunoblotting results showed that lovastatin increased the γH2AX levels dose-dependently (Figure 2C and Figure S1A). Furthermore, the treatment of HCT116 cells with 10 μM lovastatin for 6, 12, 24, and 48 h resulted in a time-dependent increase in γH2AX levels (Figure 2D and Figure S1B). Under oxidative stress, guanine will be oxidized at position 8 to generate 8-hydroxylated guanine species, serving as oxidative DNA damage markers [44]. Among these species, 8-oxoguanine (8-oxoG) and 8-hydroxydeoxyguanosine (8-OHdG) are the common biomarkers of oxidative stress. Following lovastatin treatment, 8-oxoG increased substantially in HCT116 cells as measured by an immunofluorescence method (Figure 2E). Additionally, 8-OHdG content was determined using the ELISA Kit, which demonstrated a significant increase in the statin treatment group, as compared to the control group (Figure 2F). Considering that both mtDNA integrity and mtDNA abundance were significantly reduced in statin-treated cells (Figure 1D,E), we speculated that lovastatin-induced DNA damage could be localized to mitochondria. Immunochemistry staining using MitoTracker Red CMXRos with a confocal laser scanning microscope confirmed that 8-oxoG was localized in mitochondria in lovastatin-treated HCT116 cells (Figure 2G). It has been reported that upon DNA damage, γH2AX accumulated in the cytoplasm [45,46], whereas H2AX was localized in the mitochondria [47]. Hence, we further examined the γH2AX levels in mitochondria using subcellular fractionation isolated by using the Qproteome Mitochondria Isolation Kit. HCT116 cells receiving vehicle and lovastatin treatment were separated into cytoplasmic and mitochondrial fractions, and γH2AX levels were analyzed by immunoblotting using cytoplasmic (GAPDH) and mitochondrial (Tom20) markers to monitor the purity of the fractions. After lovastatin treatment, γH2AX levels increased in both cytoplasmic and mitochondrial fractions, although it was found in the cytoplasm at higher levels (Figure 2H and Figure S1C). Collectively, these results showed that lovastatin treatment leads to the generation of ROS, resulting in DNA damage, including the impairment to mtDNA.

### 3.3. Mitochondria ROS Generation Contributes to Lovastatin-Induced DNA Damage in HCT116 Cells

**Figure 3:**
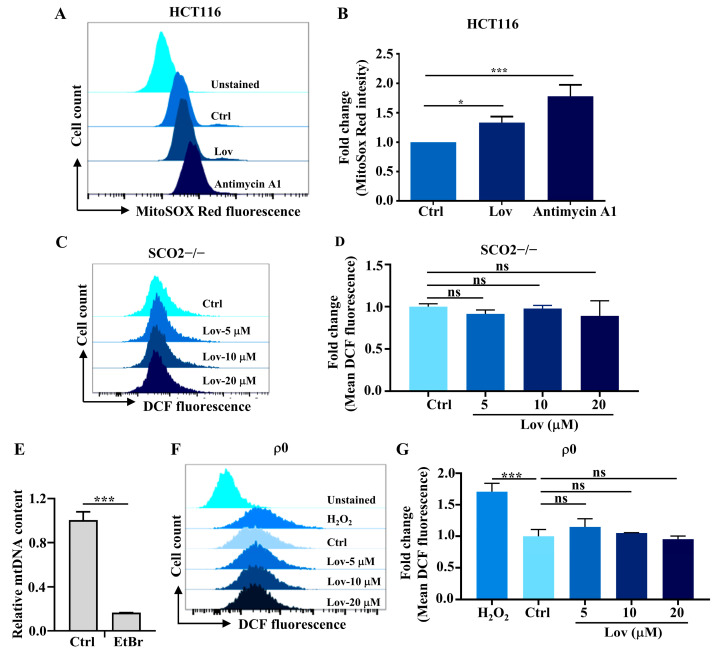
Lovastatin exposure induces mitochondrial ROS generation. (**A**) HCT116 cells were treated with 10 μM lovastatin for 48 h, or with 20 μM Antimycin A (positive control) for 40 min before staining with MitoSOX red and analyzed by flow cytometry. (**B**) Quantitative analysis of MitoSOX Red fluorescence intensities of (**A**). (**C**) After treatment with a different concentration of lovastatin for 48 h in SCO2−/− cells, the ROS production was assessed by monitoring the changes in the fluorescence intensity of DCF by flow cytometry. (**D**) Quantification data of DCF fluorescence intensity of (**C**). (**E**) Quantitative PCR analysis of mtDNA depletion from HCT116 treated with ethidium bromide (EtBr) over three weeks. (**F**) After treatment with a different concentration of lovastatin for 48 h in ρ^0^ HCT116 cells, the ROS production was assessed by monitoring the changes in the fluorescence intensity of DCF by flow cytometry. (**G**) Quantification data of DCF fluorescence intensity of (**F**). All data were obtained from at least three independent experiments. * *p* < 0.05; *** *p* < 0.01; and ns, no significance.

Endogenous ROS are produced by various organelles, including mitochondria, the NADPH oxidase complex, peroxisomes, and the endoplasmic reticulum. The primary source, however, is the mitochondria, which is responsible for approximately 90% of total cellular ROS production, thereby contributing to an extensive array of mitochondrial ROS-induced damages [48,49]. To explore whether lovastatin-induced ROS emanate from mitochondria specifically, we examined mitochondria-derived ROS using MitoSOX red via flow cytometry. Consistent with earlier studies [14,50], a significant increase in MitoSOX red fluorescence was observed within the lovastatin treatment group when compared to the control group (Figure 3A,B). The HCT116 SCO2−/− cell line, deficient in the synthesis of cytochrome c oxidase 2 (SCO2), was derived from HCT116 cells, and exhibited no mitochondrial respiration as previously described [51]. As expected, no change in the ROS production was observed in SCO2−/− cells following lovastatin treatment (Figure 3C,D). To further support this finding, we established an mtDNA-deficient HCT116 cell line (referred to as ρ^0^) by treating these cells with a low dose of ethidium bromide (EtBr) following previous protocols [34] (Figure 3E). Similar to the DCF-staining analysis in SCO2−/− cells, the production of ROS remained unaffected in ρ^0^ cells, regardless of lovastatin dosages. On the contrast, the positive control group (cells treated with 100 μM H_2_O_2_ for 4 h) showed a significant increase in ROS production (Figure 3F,G).

**Figure 4:**
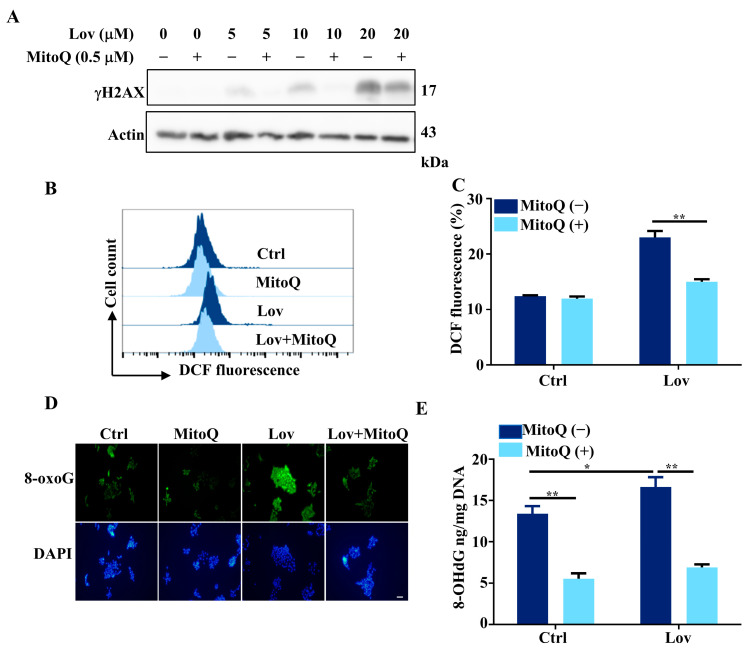
MitoQ attenuates lovastatin-induced DNA damage. (**A**) Representative immunoblotting of γH2AX in HCT116 cells treated with lovastatin in the presence or absence of 0.5 μM MitoQ (1 h prior to lovastatin) for 48 h. β-Actin served as a loading control. (**B**) HCT116 cells were treated with 10 μM lovastatin for 48 h in the presence or absence of MitoQ (0.5 μM, 1 h prior to lovastatin), and ROS production was assessed by monitoring the changes in the fluorescence intensity of DCF by flow cytometry. (**C**) Quantitative analysis of DCF fluorescence intensities of (**B**). (**D**) HCT116 cells were treated with lovastatin (10 μM) for 48 h in the presence or absence of MitoQ (0.5 μM, 1 h prior to lovastatin). Furthermore, 8-oxoG was detected by immunofluorescence staining. Bar, 50 μm. (**E**) The content of 8-OHdG was quantified using 8-OHdG ELISA enzyme-linked immunosorbent assay in HCT116 cells treated with 10 μM lovastatin for 48 h in the presence or absence of MitoQ (0.5 μM, 1 h prior to lovastatin). All data were obtained from at least three independent experiments. * *p* < 0.05, ** *p* < 0.01.

The mitochondrial-targeted ubiquinone, MitoQ, is a lipophilic triphenylphosphonium cation covalently attached to an ubiquinol antioxidant, acting as an effective mitochondrial-targeted ROS scavenger [52]. To assess whether DNA damage induced by lovastatin could be attenuated by MitoQ, we examined the γH2AX levels in HCT116 cells treated with lovastatin at serial concentrations (0, 5, 10, 20 μM) with or without MitoQ. Consistent with the results indicated in Figure 2C, lovastatin induced γH2AX levels in a dose-dependent manner (Figure 4A and Figure S2). However, γH2AX levels were notably reduced in the group treated with both MitoQ and lovastatin, compared to the group treated with lovastatin alone. To further evaluate whether MitoQ could attenuate ROS production in HCT116 cells treated with lovastatin, we measured the ROS levels using DCF staining and proceeded with flow cytometry. Consistent with a previous study [53], MitoQ did not affect ROS production in the control group (Figure 4B,C). However, intracellular ROS production was significantly reduced by MitoQ in lovastatin-treated HCT116 cells (Figure 4B,C), suggesting that lovastatin-induced ROS production primarily originates from the mitochondria. Notably, the immunofluorescence assay demonstrated that the fluorescence level of 8-oxoG was substantially mitigated by MitoQ in the lovastatin-treated group (Figure 4D). In accordance with the reduction of 8-oxoG, we also recorded a decrease in 8-OHdG content in HCT116 cells treated with both MitoQ and lovastatin (Figure 4E). Taken together, these results collectively support the notion that the ROS generation induced by lovastatin stems predominantly from the mitochondria, and the mitochondrial-targeted ROS scavenger MitoQ significantly attenuates lovastatin-induced DNA damage in HCT116 cells. 

### 3.4. Lovastatin Promotes Accumulation of Cytosolic mtDNA and Activates cGAS-STING Pathway

**Figure 5:**
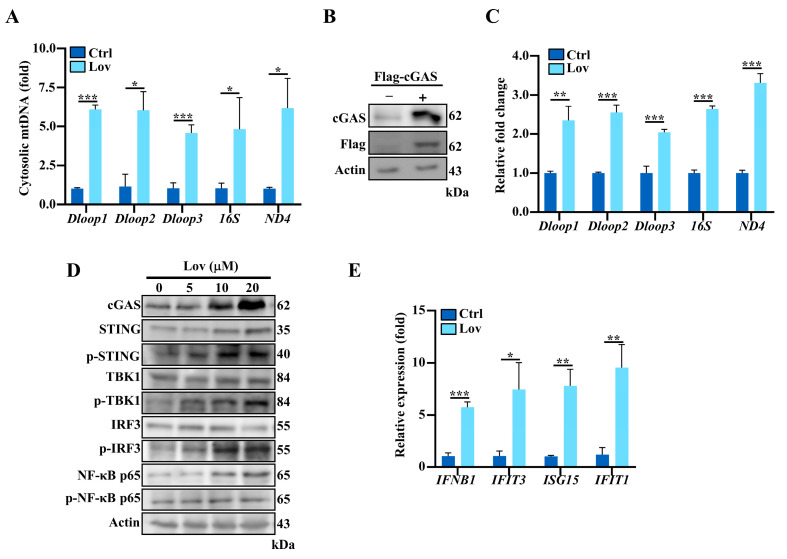
Lovastatin promotes accumulation of cytosolic mtDNA and activates cGAS-STING pathway. (**A**) HCT116 cells were treated with 10 μM lovastatin for 48 h; cytosolic mtDNA was quantified by qPCR using mitochondrial gene primer sets. Normalization was performed by using *18S rRNA*. (**B**) Western blot showing the stable expression of Flag-cGAS in HCT116 cells. β-Actin served as a loading control. (**C**) The cytosolic mtDNA was quantified by qPCR in HCT116 Flag-cGAS stable cells treated with 10 μM lovastatin for 48 h. (**D**) Representative immunoblots of cGAS, STING, p-STING, TBK1, p-TBK1, IRF3, p-IRF3, NF-κB p65, and p-NF-κB p65 from HCT116 treated with a serial concentration of lovastatin for 48 h. β-Actin served as a loading control. (**E**) qPCR analysis of the expression of *ISG15*, *IFIT3*, *IFIT1*, and *IFNB1* in HCT116 cells treated with 10 μM lovastatin for 48 h. All data were obtained from at least three independent experiments. * *p* < 0.05, ** *p* < 0.01, *** *p* < 0.001.

Since mitochondrial stress can trigger the release of mtDNA into the cytosol, we wondered whether the oxidative stress induced by lovastatin could also trigger mtDNA release into the cytosol. To detect cytosolic mtDNA, we performed a qPCR to analyze the relative mtDNA amount in the cytosolic extracts, as previously described [38]. The results revealed a four-to-six-fold increase in specific mtDNA fragments from the D-loop regulatory region, *16S rRNA,* and NADH dehydrogenase protein subunit 4 (*ND4*), indicating the liberation of mtDNA into the cytosol (Figure 5A). Since the accumulation of cytosolic mtDNA could be recognized by the DNA sensor cGAS, and the activation of the cGAS-STING pathway resulted in IRF3 activation and an increase in a type I IFN response [54,55], we ascertained a lovastatin-induced mtDNA leakage into the cytosol and binding to cGAS. Flag-tagged cGAS from HCT116 cells treated with lovastatin was immunoprecipitated, followed by a qPCR for the detection of mtDNA (Figure 5B and Figure S3A). The results showed that, in response to lovastatin treatment, cGAS was bound to mtDNA with evidence of a two-to-three-fold increase of D-loop regulatory region, *16S rRNA*, and *ND4* (Figure 5C). To further investigate whether the cGAS-STING pathway signaling was activated by lovastatin, we examined the protein expression levels of cGAS, STING, TBK1, and IRF3, as well as their phosphorylated forms. As shown in Figure 5D and Figure S3B, lovastatin significantly increased cGAS expression in a dose-dependent manner. Meanwhile, a significant increase in the levels of phosphorylated STING was also observed (Figure 5D and Figure S3C). STING activation can also trigger downstream nuclear factor kB (NF-κB) [56]. The immunoblotting results confirmed that the phosphorylation level of NF-κB was increased in the lovastatin treatment cells (Figure 5D and Figure S3G). Additionally, it is well known that TBK1 and IRF3 are two important factors of multiple anti-viral signaling pathways, including cytosolic DNA sensor signaling through the cGAS-STING signaling pathway [57]. Consistently, the phosphorylated levels of TBK1 and IRF3 were both dose-dependently increased in lovastatin-treated cells, as demonstrated in Figure 5D and Figure S3E,F. Since phosphorylated IRF3 subsequently dimerizes and translocates to the nucleus and then induces the transcription of type I IFNs [18], we assessed the effect of lovastatin on type I IFNs mRNA expression levels using a qPCR. Compared to the control group, the lovastatin-treated group had five-to-nine-fold elevated transcripts for the type I IFNs gene, including *IFNB1*, *IFIT3*, *ISG15*, and *IFIT1*. Finally, these findings collectively show that lovastatin triggers the release of mtDNA into the cytosol and activates the cGAS-STING pathway in HCT116 cells.

### 3.5. Type 1 IFNs Signaling Induced by Lovastatin Is Dependent on cGAS/STING

**Figure 6:**
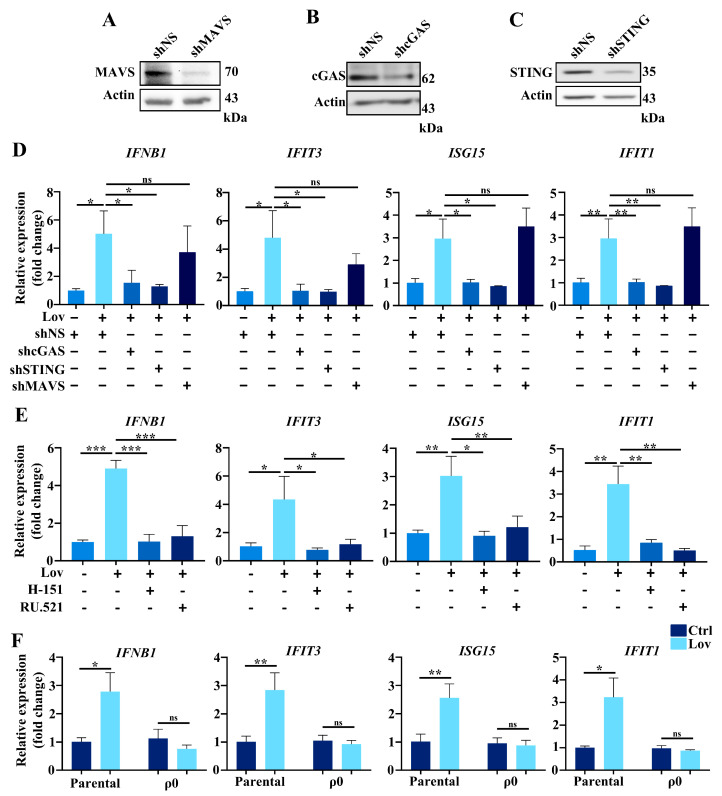
Type 1 IFN signaling induced by lovastatin is dependent on cGAS/STING. (**A**) Western blotting analysis of MAVS after lentiviral knockdown in HCT116 cells. β-Actin served as a loading control. (**B**) Western blotting analysis of cGAS after lentiviral knockdown in HCT116 cells. β-Actin served as a loading control. (**C**) Western blotting analysis of STING after lentiviral knockdown in HCT116 cells. β-Actin served as a loading control. (**D**) The expressions of *IFNB1, IFIT3*, *ISG15*, *IFIT1* were quantified by qPCR in cGAS, STING, and MAVS knockdown cells treated with 10 μM lovastatin for 48 h. (**E**) HCT116 cells were pretreated either with STING inhibitor H-151 (1.5 μM) or cGAS inhibitor RU.521 (1.0 μM) for 2 h, and afterward treated with lovastatin for 24 h, the expressions of *IFNB1, IFIT3*, *ISG15*, *IFIT1* were quantified by qPCR. (**F**) The expressions of *IFNB1*, *IFIT3*, *ISG15*, *IFIT1* were quantified by qPCR in parental and ρ^0^ HCT116 cells treated with 10 μM lovastatin for 48 h. All data were obtained from at least three independent experiments. * *p* < 0.05; ** *p* < 0.01; *** *p* < 0.001; and ns, no significance.

The activation of different signaling modules such as RIG-I/MDA5 (retinoic acid-inducible gene-1, RIG-I; melanoma differentiation-associated protein 5, MDA5), cGAS-STING, and TLR3/4-TRIF (TLR3/4, Toll-like receptors 3/4) results in the production of type I IFNs [18]. To identify the sensor which regulates lovastatin-induced type I IFNs (*IFNB1*, *IFIT3*, *ISG15*, and *IFIT1)*, the conserved signaling adaptors MAVS, cGAS, or STING were knocked down in HCT116 cells, and a Western blot was conducted to confirm gene knockdown efficacy by shRNA (Figure 6A–C and Figure S4). As shown in Figure 6D, the absence of MAVS did not reduce the activation of type I IFNs induced by lovastatin. Instead, the knockdown of cGAS, a sensor of cytosolic DNA, reduced the activation of type I IFNs to baseline (Figure 6D). Once cGAS senses cytosolic DNA, it will in turn activate STING to upregulate the expression of type I IFNs. Consistently, we then observed a significant attenuation of the expression levels of *IFNB1*, *IFIT3*, *ISG15*, and *IFIT1* in STING knockdown HCT116 cells treated with lovastatin (Figure 6D). In a complementary approach, we evaluated the gene expressions of lovastatin-induced type I IFNs by blocking the pathway with the pharmacological inhibitor of cGAS (RU.521) and STING (H-151). Indeed, these two inhibitors both prevented the expression of *IFNB1*, *IFIT3*, *ISG15*, and *IFIT1* in response to lovastatin treatment (Figure 6E). Since cGAS can respond to double-stranded DNA (dsDNA) of either nuclear or mitochondrial origin [58,59], to ascertain the source of DNA activating cGAS in response to lovastatin treatment, we next examined whether type I IFNs expression could be influenced in ρ^0^ cells. The results demonstrated that unlike in the parental cells, the expression levels of *IFNB1*, *IFIT3*, *ISG15*, and *IFIT1* were not increased in ρ^0^ cells treated with lovastatin (Figure 6F). As shown in Figure 4C, cytosolic mtDNA bound to cGAS after lovastatin treatment in HCT116 cells. Collectively, we concluded that lovastatin caused mtDNA to be released to the cytosol by promoting cGAS/STING activation.

### 3.6. Lovastatin-Induced Apoptosis Partially Mediated by cGAS-STING Pathway in HCT16 Cells

**Figure 7:**
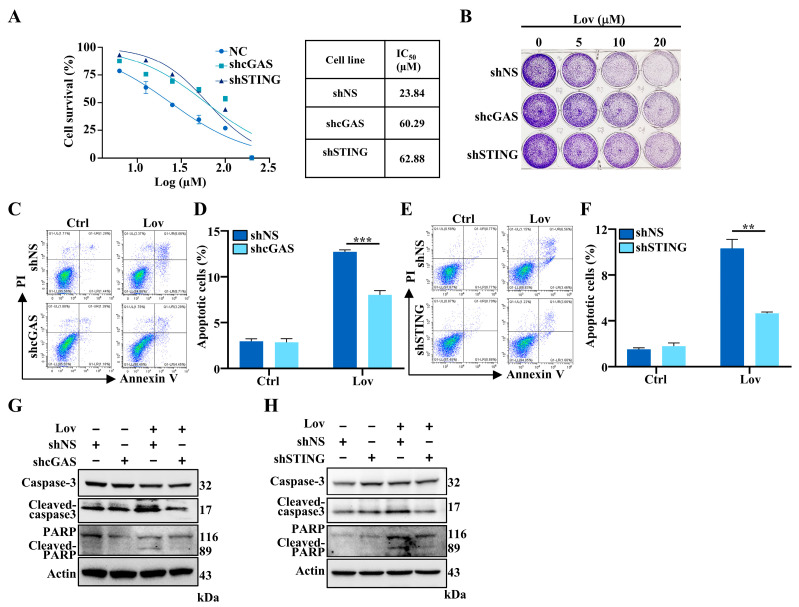
cGAS-STING is associated with lovastatin-induced apoptosis in HCT116 cells. (**A**) Measurement of lovastatin IC_50_ of cGAS, STING knockdown and parental HCT116 cells by SRB assay. (**B**) Crystal violet cell viability staining of cGAS and STING knockdown HCT116 cells after treatment with indicated concentration of lovastatin for 72 h. (**C**) After treatment with 10 μM lovastatin for 48 h in cGAS knockdown HCT116 cells, apoptosis was determined by Annexin V and PI staining. (**D**) Quantification data of the apoptotic cells. (**E**) After treatment with 10 μM lovastatin for 48 h in STING knockdown HCT116 cells, apoptosis was determined by Annexin V and PI staining. (**F**) Quantification data of the apoptotic cells. (**G**) Representative immunoblots of caspase-3, cleaved-caspase-3, PARP, cleaved-PARP from control (shNS), and cGAS knockdown HCT116 cells treated with 10 μM lovastatin for 48 h. β-Actin served as a loading control. (**H**) Representative immunoblots of caspase-3, cleaved-caspase-3, PARP, cleaved-PARP from control (shNS), and STING knockdown HCT116 cells treated with 10 μM lovastatin for 48 h. β-Actin served as a loading control. All data were obtained from at least three independent experiments. ** *p* < 0.01, *** *p* < 0.01.

The cGAS-STING pathway can be activated by DNA damage [57,58]. In this study, we demonstrated that lovastatin induced DNA damage, leading to mtDNA leakage into the cytosol. This activated the cGAS-STING pathway and downstream activation of type I IFNs. Previous research, including work performed by our group, has shown that lovastatin induced apoptosis in CRCs [17]. We subsequently aimed to illustrate whether the cGAS-STING pathway was involved in lovastatin-induced apoptosis in HCT116 cells. To determine the effect of cGAS-STING on the viability of HCT116 cells treated with lovastatin, we subjected cGAS- and STING knockdown cells to lovastatin. The knockdown of either cGAS or STING could reverse the cytotoxic effects of lovastatin at low concentrations. However, it was only partly attenuated at high concentrations (Figure 7A,B). The results indicated that the knockdown of both cGAS and STING mitigated the growth inhibitory effect of lovastatin (Figure 7A). The IC_50_ of lovastatin was 60.29 µM and 62.88 µM, respectively, in cGAS- and STING knockdown cells, which was higher than the IC_50_ of 23.84 µM in the shNS group. Further, we performed crystal violet staining to analyze cell viability post-lovastatin treatment. Consistently, both the cGAS and STING knockdown decreased the rate of lovastatin-induced cell death (Figure 7B). The results were confirmed by measuring apoptosis in lovastatin-treated cells using flow cytometry. The knockdown of either cGAS or STING did not affect apoptosis in HCT116 cells, consistent with a previous study [31]. Lovastatin treatment resulted in a 10.30% increase in Annexin V-positive fractions of shNS HCT116 cells, but a 5.66% decrease was observed in STING knockdown cells post-lovastatin treatment compared to the control group (Figure 7E,F). Similar results were observed in cGAS knockdown cells (Figure 7C,D). Finally, we confirmed these results by immunoblotting, which demonstrated a decrease in the cleaved forms of caspase-3 and PARP, protein markers of apoptosis, in cGAS and STING knockdown cell post-lovastatin treatment (Figure 7G,H and Figure S5).

**Figure 8:**
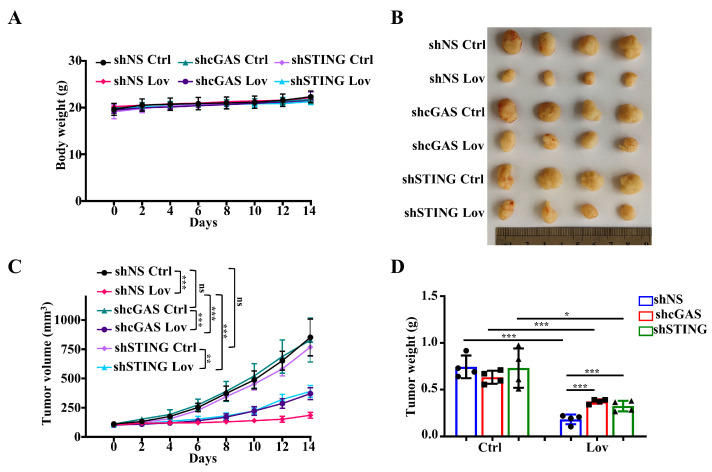
Knockdown of STING or cGAS attenuates the antitumor effect of lovastatin in HCT116 cells. shNS, STING knockdown, or cGAS knockdown HCT116 cells were injected subcutaneously into nude mice and the mice were intraperitoneally administered 20 mg/kg lovastatin daily for two weeks. (**A**) Animal weight of each treatment group. (**B**) Tumor volume of each treatment group. (**C**) Tumors excised from mice in (**B**). (**D**) Tumor weight of each treatment group in all animals that were sacrificed after 14 days of treatment. Data were shown as the means ± SD (*n* = 4). * *p* < 0.05; ** *p* < 0.01; *** *p* < 0.01; and ns, no significance.

Finally, to extend the in vitro results, the antitumor effect of lovastatin was measured in STING or cGAS knockdown HCT116 tumor-bearing nude mice. As shown in Figure 8A, there was no significant difference in body weight among all mice in each treatment group. Consistent with previous studies [60], lovastatin treatment significantly reduced the tumor volume and weight in shNS HCT116-bearing mice compared to the control group (Figure 8B–D). The knockdown of STING or cGAS did not affect the tumor volume and tumor weight in the control group (Figure 8B–D), which is consistent with a previous study [61]. Notably, the antitumor effect of lovastatin was partially attenuated in STING knockdown HCT116 tumor-bearing mice, as evidenced by a moderate but not complete reversal of the antitumor effect in terms of tumor volume and weight (Figure 8B–D). Accordingly, we also observed the same results in cGAS knockdown HCT116 tumor-bearing mice. Overall, these in vitro and in vivo data show that the cGAS/STING pathway is involved in the process of apoptosis, and inhibition of this pathway partially reduces the cytotoxicity of lovastatin in HCT116 cells in vitro and in vivo.

## 4. Discussion

Statins, inhibitors of HMG-CoA reductase, are renowned for their effective cholesterol-lowering properties. Both in vitro and in vivo studies suggest that statins have a growth inhibitory effect and induce apoptosis across a variety of cancer cells [62], though the anticancer mechanisms remain incompletely understood. In this study, we explored the effect of lovastatin on promoting oxidative DNA damage in HCT116 cells and inducing apoptosis through activation of the cGAS-STING pathway. In line with our previous work [17], we demonstrated that lovastatin treatment leads to mitochondrial dysfunction (Figure 1) by depolarizing the mitochondrial membrane, reducing the OCR, and decreasing mtDNA integrity and content. Importantly, mitochondrial dysfunction, corresponding to elevated mitochondrial ROS production (Figure 2 and Figure 3) and mtDNA damage (Figure 2G,H), was also induced by lovastatin treatment. This was attenuated by the mitochondria targeting antioxidant mitoQ (Figure 4). Mechanistically, mtDNA damage resulted in cytosolic mtDNA accumulation (Figure 5A), leading to recognization by cGAS (Figure 5B,C), thereby activating the cGAS-STING pathway (Figure 5D,E). The elevated expression of type I IFN was dependent on the mtDNA-activated cGAS-STING pathway (Figure 6). Furthermore, we found that the activation of the cGAS-STING pathway played a role in lovastatin-induced apoptosis, as the cytotoxicity (Figure 7A,B) and apoptosis (Figure 7C–H) were both partially mitigated in both cGAS and STING knockdown cells. Moreover, the antitumor effect of lovastatin was also attenuated by the knockdown of cGAS or STING in the HCT116 xenograft model in vivo (Figure 8).

Over the decades, statins have been reported to exhibit numerous pleiotropic effects. The suppression of global oxidative stress is hypothesized to be one of the most significant mechanisms through which they exert beneficial effects on the cardiovascular system [63,64]. However, accumulating evidence also shows that statins can increase oxidative stress in other organs or tissues [16]. This oxidative stress has been implicated as the cause of the adverse effects of statins, such as myopathy [65], various diabetic complications [66], and the development of fatty liver [67]. Therefore, the effects of statins on oxidative stress appear to be dependent on specific cell types, tissues, and organs [68]. Coenzyme Q10 (CoQ10), a metabolite of the mevalonate pathway and an essential cofactor for electron transport, resides in the inner mitochondrial membrane. It is not only an essential electron carrier between complex I or II and complex III but also serves as an important antioxidant [69]. The current study demonstrated that lovastatin induced mitochondrial ROS production (Figure 3) and mtDNA damage (Figure 2G,H) in HCT116 cells. Furthermore, mitoQ, one of the most well-characterized mitochondria-targeted antioxidants which consists of a CoQ10 moiety conjugated to a triphenylphosphonium (TPP+) cation to facilitate accumulation within the mitochondria, significantly attenuated lovastatin-induced mitochondrial ROS production and DNA damage (Figure 4) [70]. Notably, γH2AX was detected both in the cytoplasm and mitochondria (Figure 2H). While γH2AX is commonly found in the nucleus, studies have also reported its accumulation in the cytoplasm during DNA damage induced by the overactivation of tropomyosin receptor kinase A (TrkA) in the human osteosarcoma U2OS cell line [46,71], and after bee venom treatment in liver and breast cancer cells [55]. Additionally, Jeong et al. also reported the mitochondrial localization of H2AX [47]. Therefore, the cellular pathway responsible for λH2AX protein accumulation in the cytoplasm and mitochondria following lovastatin treatment in HCTT116 requires further investigation.

Studies have shown that the abundance of the cGAS protein and the cGAS-STING signaling varies in different cell lines [72,73,74,75,76,77]. In fact, the level of cGAS expression in HCT116 cells has been controversial, with some studies reporting its presence [72,73,74,77] and others failing to detect it [75,76]. Our results showed that the cGAS protein was detected along with a functional cGAS-STING pathway in HCT116 cells, which was activated by lovastatin (Figure 5D,E). Accumulating evidence has shown that the expression of the cGAS-STING pathway is associated with its methylation level, which is mediated by two types of enzymes, DNA methyltransferase (DNMT) and DNA demethylase [78,79]. Statins were reported to serve as DNMT inhibitors to augment the chemosensitivity of CRCs by inducing epigenetic reprogramming and reducing CRC stemness via the bone morphogenetic protein pathway [80]. Hence, we speculated that the increased expression level of cGAS caused by lovastatin treatment may have partly contributed to the inhibition of DNMT (Figure 5D).

Importantly, the intrinsic DNA damage caused by cytoplasmic dsDNA accumulation, originating from both genomic DNA and mtDNA, activates the cGAS-STING pathway [81]. Our data demonstrated that cGAS binded to mtDNA which was released into the cytosol and activated the cGAS-STING pathway in HCT116 cells treated with lovastatin (Figure 5 and Figure 6). Although mitochondria double-stranded RNA (mtdsRNA) can also trigger a type I IFN response through a MDA5-driven antiviral signaling pathway [28], our results showed that mtdsRNA did not contribute to the expression of type I IFN (Figure 6E). Although oxidative DNA damage was also detected in nuclear DNA in this study, mtDNA is more sensitive to oxidative damage than nuclear DNA and mtDNA damage takes longer to repair [9,82]. Unlike normal cells, cancer cells are often replete with cytosolic dsDNA. Although this dsDNA can derive from genomic, mitochondrial, and exogenous origins [81], recent evidence suggests that the primary source is chromosomal instability (CIN), a hallmark of cancer [81]. Due to unstable genomes in cancer cells, chromosomes are more susceptible to missegregate into micronuclei during mitosis (47). Moreover, chromosomes enclosed in a micronuclei experience increase DNA damage and can be exposed to the cytosol upon micronuclear envelope rupture [83,84]. While the activation of cGAS-STING signaling in tumors might provide a powerful means to mobilize immune surveillance leading to tumor clearance, tumors with CIN will likely adapt to sustained cGAS-STING signaling and become insensitive to nuDNA. Notably, a previous study reported that both complex inhibitor rotenone and the mutational inactivation of Cockayne syndrome B (CSB) stimulate intrinsic mitochondrial ROS production and cause oxidative damage in mtDNA, rather than to the oxidative nuclear DNA damage [9,85,86]. Consistent with this finding, we observed oxidative mtDNA damage in HCT116 cells treated with lovastatin (Figure 4) and the failure to detect cGAS-STING upregulated type I IFNs expression in mtDNA-depleted HCT116 ρ^0^ cells (Figure 6E). 

The cGAS-STING pathway not only facilitates inflammatory responses and the production of type I IFNs but also activates other cellular processes, such as apoptosis through the regulation of ER stress [87,88], NOD-like receptor 3 (NLRP3) [89], IRF3 [90], and IFN signals [91,92]. Moreover, cGAS-STING-dependent apoptosis is also reported to be mediated by NF-κB, a major component in the DNA damage pathway NF-κB [31,93]. Our results also showed that NF-κB was activated by lovastatin (Figure 5). Recently, a study reported that lovastatin can activate the STING pathway in an Src homology-2 domain-containing protein tyrosine phosphatase-2 (SHP2)-dependent manner to partially reverse the chemoresistance process by apoptosis induction and cell-cycle arrest in CRCs [94]. Here, we observed that cGAS-STING activation by cytosolic mtDNA contributed, at least partially, to apoptosis induced by lovastatin in HCT116 cells (Figure 7). However, the underlying mechanisms of how lovastatin induced apoptosis through activating the cGAS-STING pathway are not fully understood. Therefore, its mechanisms in human CRC HCT116 cells will be the subject of future studies.

Notably, the results of this study should be interpreted by considering the doses of lovastatin used in the experiment. As reviewed by Stamm et. al. although statin concentrations in human serum are typically in the nM range, the concentrations used in in vitro cancer research range from 1 to 200 μM [95] and their accumulation within tumor tissues remains unclear. For the cell viability assay in Figure 7A, the IC_50_ value of lovastatin was 23.84 μM, which may vary between cell lines. For animal studies, the dose of 20 mg/kg daily was used based on previous studies [96,97]. Pecoraro and co-workers summarized that the doses of statins used in the majority of rodent studies range between 1 and 100 mg/kg, significantly higher than those prescribed to patients [98]. However, the discrepancy in dosages between humans and rodents likely reflects a pharmacodynamic resistance to the pharmacological effects of statins observed specifically in rodents [99].

The most frequent adverse reactions affecting the skeletal muscle associated with statin usage are statin-associated muscle symptoms (SAMS). As reviewed by Liu et al. [16], oxidative stress could also be responsible for statin-possible adverse effects such as myopathy. In this study, we demonstrated that lovastatin activates the cGAS-STING pathway through oxidative stress-induced DNA damage. This signaling pathway could be continuously activated in long-term statin users, potentially leading to chronic inflammation. Therefore, our study provided a potential mechanism of SAMS. However, further investigation is needed to fully understand the role of the cGAS-STING pathway in SAMS. 

In summary, our study provides new insights into a potential mechanism of statin-induced apoptosis, which is mediated partly through oxidative mtDNA damage. This damage triggers mtDNA accumulation in the cytosol, activating the cGAS-STING pathway in CRC. However, there are several limitations associated with this study. Firstly, due to the heterogeneity typical of human tumors, it must be determined whether this phenotype is restricted to this specific cell line, or whether it can be generalized to other CRCs or different types of cancers. Additionally, since patient-derived xenograft (PDX) models more closely recapitulate the native tumor biology, tissue composition, and molecular characteristics, it is better to extend this study to PDX models. It is also important to note that a higher dose of lovastatin was used in our experiments, which might limit the direct translatability of our results into practical clinical applications. Furthermore, considering that the impact on mitochondria may vary among different statins, it is necessary to validate the effects of lovastatin by using other statins. Moreover, mitochondrial components such as the mitochondrial permeability transition pore (mPTP) and voltage-dependent anion channel 1 (VDAC1), which may participate in the release of mtDNA induced by lovastatin, were not investigated in our study. Overall, future studies should aim to address these limitations by confirming the phenotype in diverse cancer types and models, considering the high dose of lovastatin used, validating the effects with other statins, and investigating mitochondrial components like mPTP and VDAC1 involved in mtDNA release.

## Data Availability

All data used and analyzed during the current study are available from the corresponding author upon reasonable request due to data sharing agreements with collaborators.

## Supplementary Materials

The following supporting information can be downloaded at: https://www.mdpi.com/article/10.3390/antiox13060679/s1, Figure S1: Densitometric analysis of Western blots in Figure 2 by using ImageJ software version 1.54g; Figure S2: Densitometric analysis of Western blots in Figure 4A by using ImageJ software version 1.54g; Figure S3: Densitometric analysis of Western blots in Figure 5 by using ImageJ software version 1.54g; Figure S4: Densitometric analysis of Western blots in Figure 6 by using ImageJ software version 1.54g; Figure S5: Densitometric analysis of Western blots in Figure 7 by using ImageJ software version 1.54g; Table S1: Oligonucleotides used for cytosolic mtDNA detection; Table S2: Oligonucleotides used for type I IFNs detection.
