# Supplementary material for: Lovastatin-Induced Mitochondrial Oxidative Stress Leads to the Release of mtDNA to Promote Apoptosis by Activating cGAS-STING Pathway in Human Colorectal Cancer Cells
Source: Antioxidants (Basel). 2024 May 31;13(6):679. doi: 10.3390/antiox13060679 (PMC11200898; doi:10.3390/antiox13060679)
Supplement: Supplementary file 1 [file antioxidants-13-00679-s001.zip › Supplement figure legends.pdf]

Supplement figure legends

Figure S1. Densitometric analysis of western blots in Figure 2 by using ImageJ software. (A) Densitometric analysis of western blots related to Figure 2B. (B) Densitometric analysis of western blots related to Figure 2C. (C) Densitometric analysis of western blots related to Figure 2H. *p<0.05, **p<0.01, ***p<0.001.

Figure S2. Densitometric analysis of western blots in Figure 4A by using ImageJ software. **p<0.01, ***p<0.001 and ns, no significance.

Figure S3. Densitometric analysis of western blots in Figure 5 by using ImageJ software. (A) Densitometric analysis of western blots related to Figure 5B. (B-G) Densitometric analysis of western blots related to Figure 2D. *p<0.05, **p<0.01, ***p<0.001 and ns, no significance.

Figure S4. Densitometric analysis of western blots in Figure 6 by using ImageJ software. (A) Densitometric analysis of western blots related to Figure 6B. (B) Densitometric analysis of western blots related to Figure 6B. (C) Densitometric analysis of western blots related to Figure 6C. **p<0.01, ***p<0.001.

Figure S5. Densitometric analysis of western blots in Figure 7 by using ImageJ software. (A) Densitometric analysis of western blots related to Figure 7G. (B) Densitometric analysis of western blots related to Figure 7H. *p<0.05, **p<0.01 and ns, no significance.
