# Supplementary figures and images for: Lovastatin-Induced Mitochondrial Oxidative Stress Leads to the Release of mtDNA to Promote Apoptosis by Activating cGAS-STING Pathway in Human Colorectal Cancer Cells
Source: Antioxidants (Basel). 2024 May 31;13(6):679. doi: 10.3390/antiox13060679 (PMC11200898; doi:10.3390/antiox13060679)

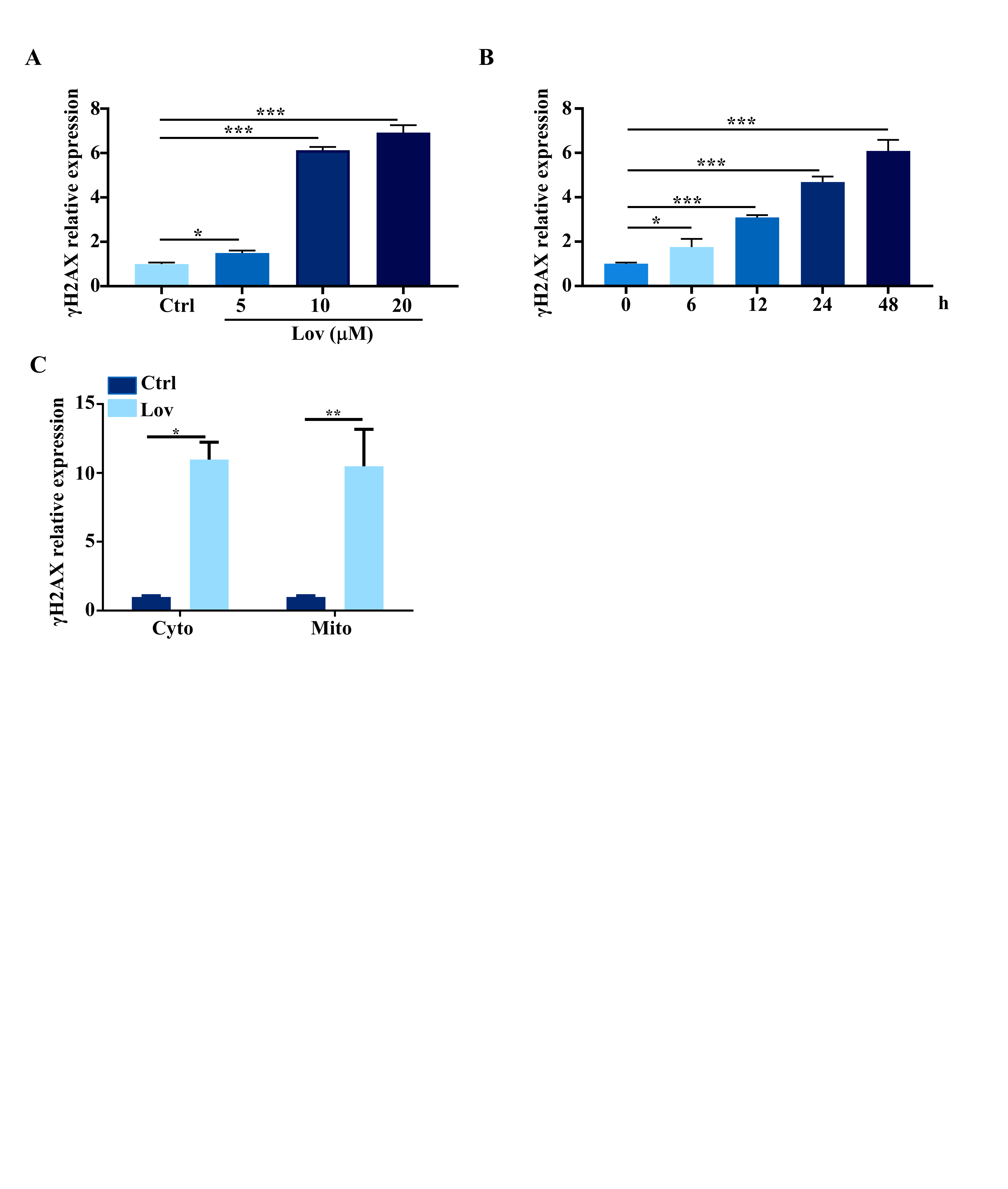

Supplement: Supplementary file 1 [file antioxidants-13-00679-s001.zip › Figure S1.tif]

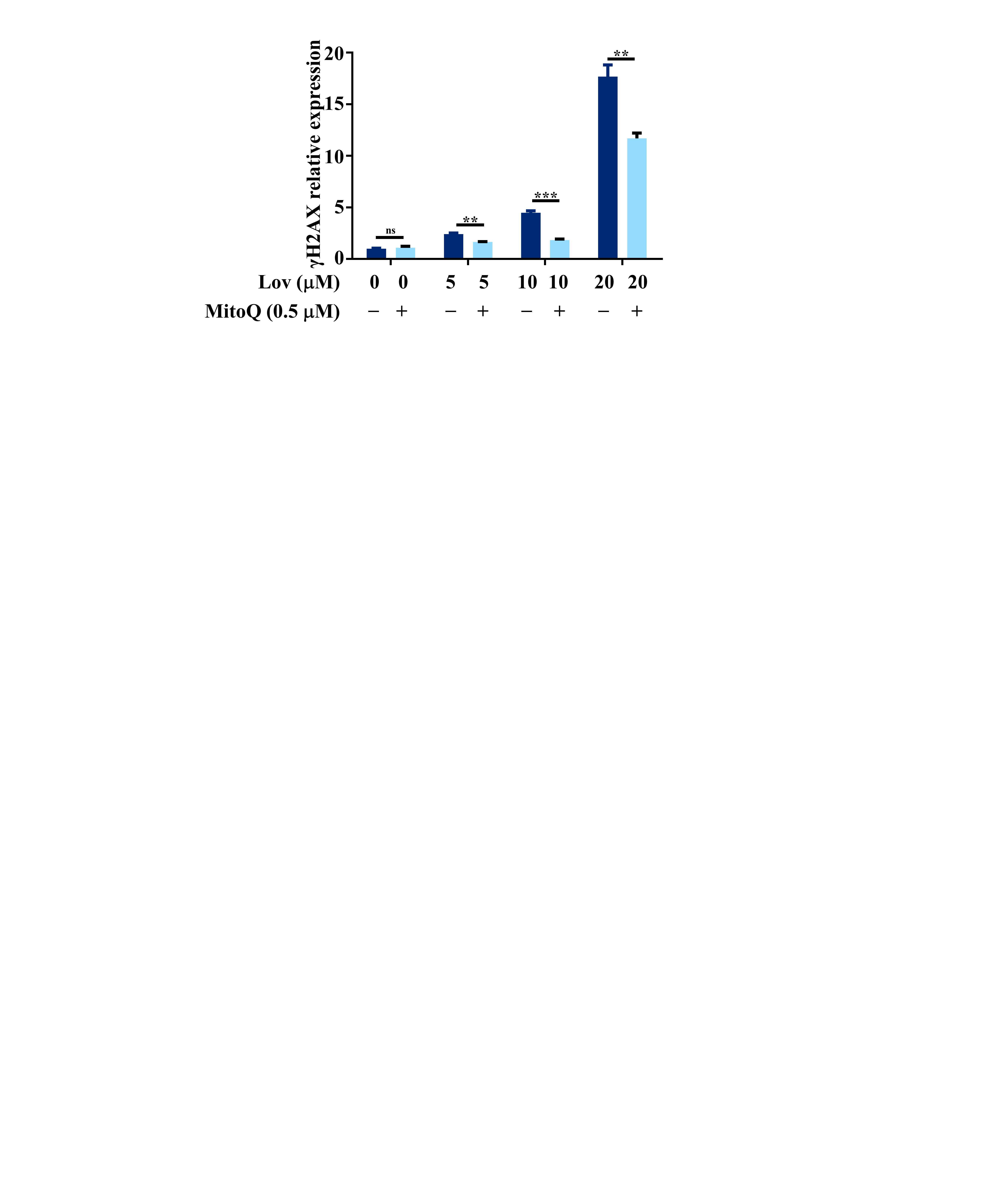

Supplement: Supplementary file 1 [file antioxidants-13-00679-s001.zip › Figure S2.tif]

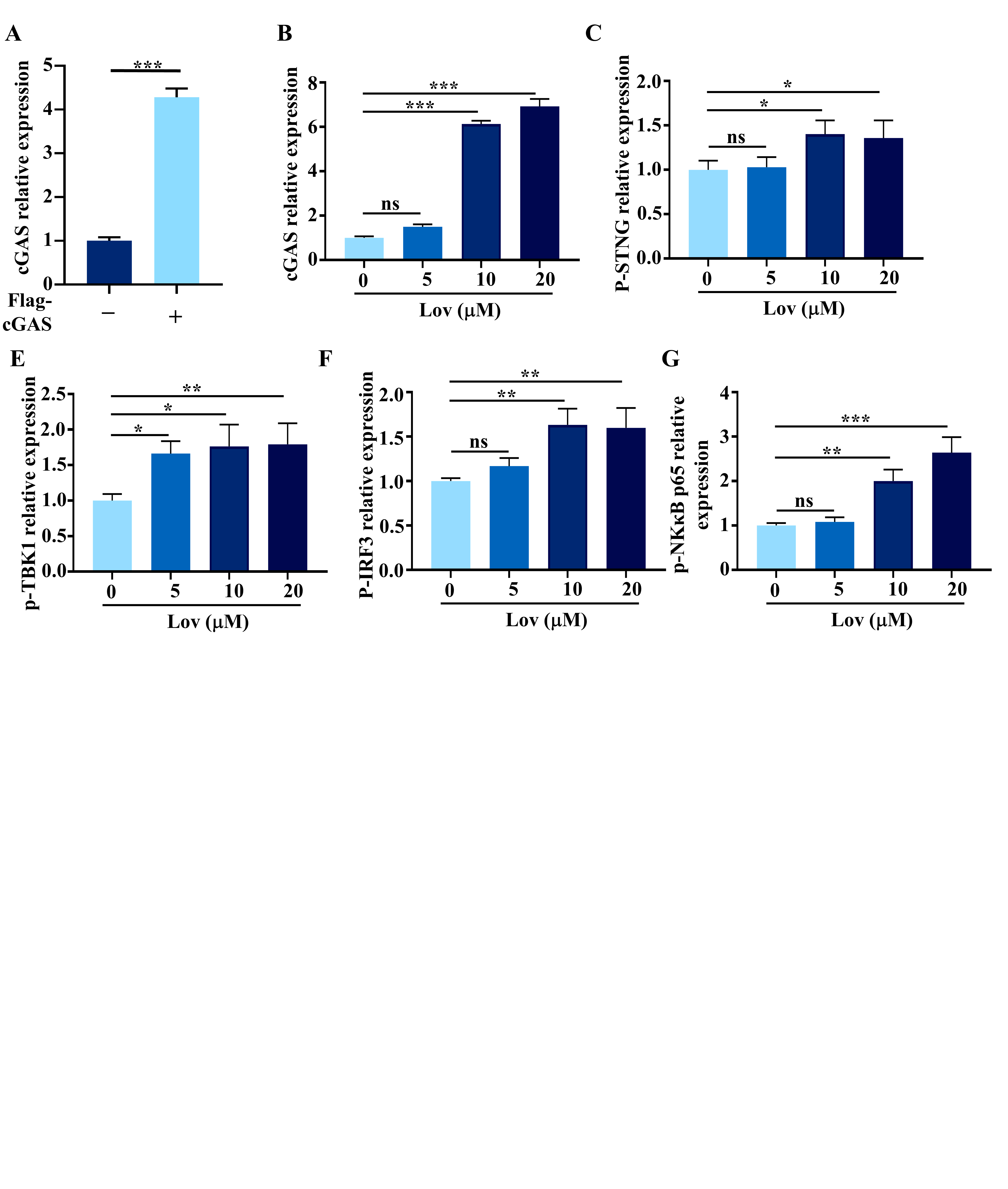

Supplement: Supplementary file 1 [file antioxidants-13-00679-s001.zip › Figure S3.tif]

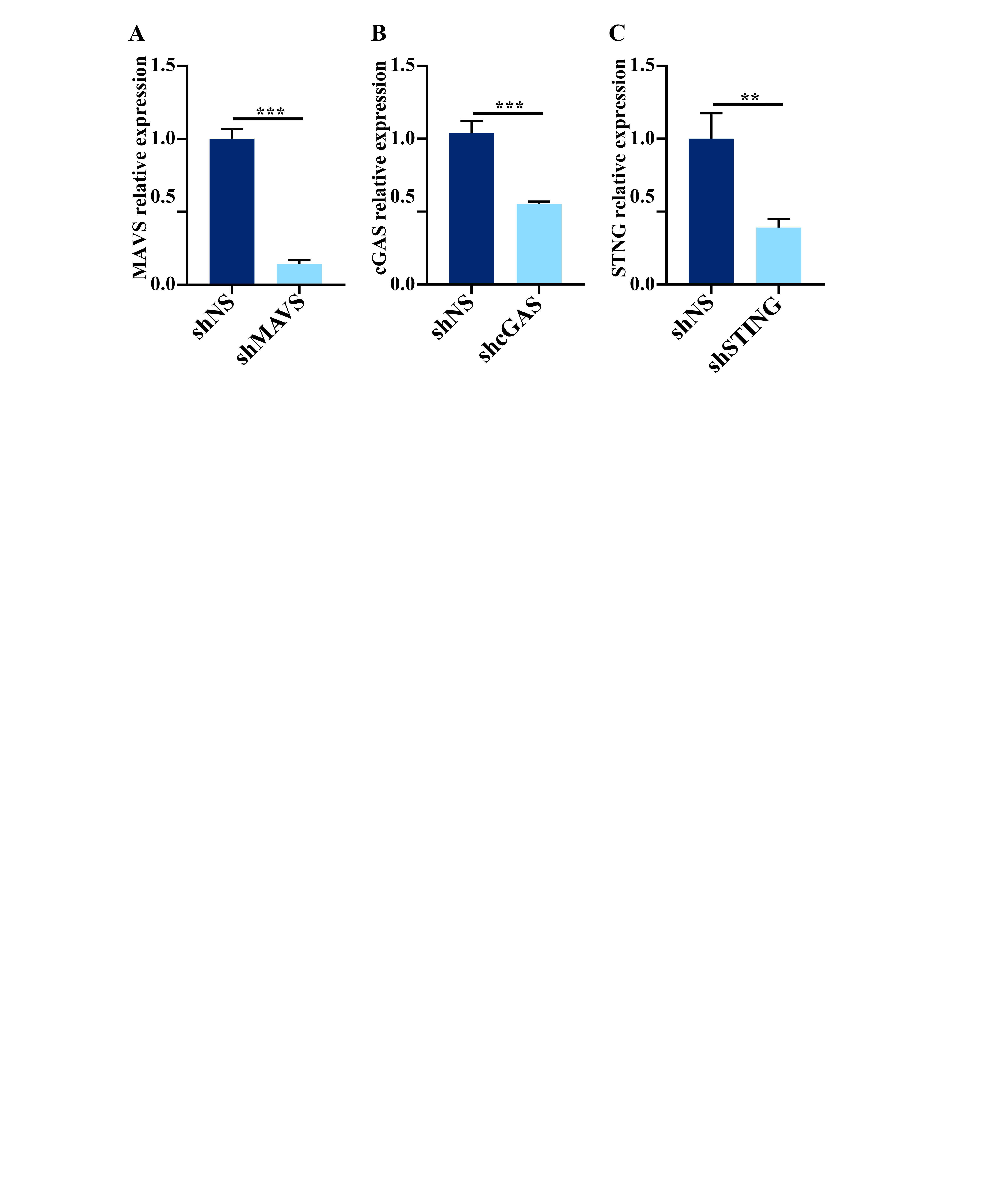

Supplement: Supplementary file 1 [file antioxidants-13-00679-s001.zip › Figure S4.tif]

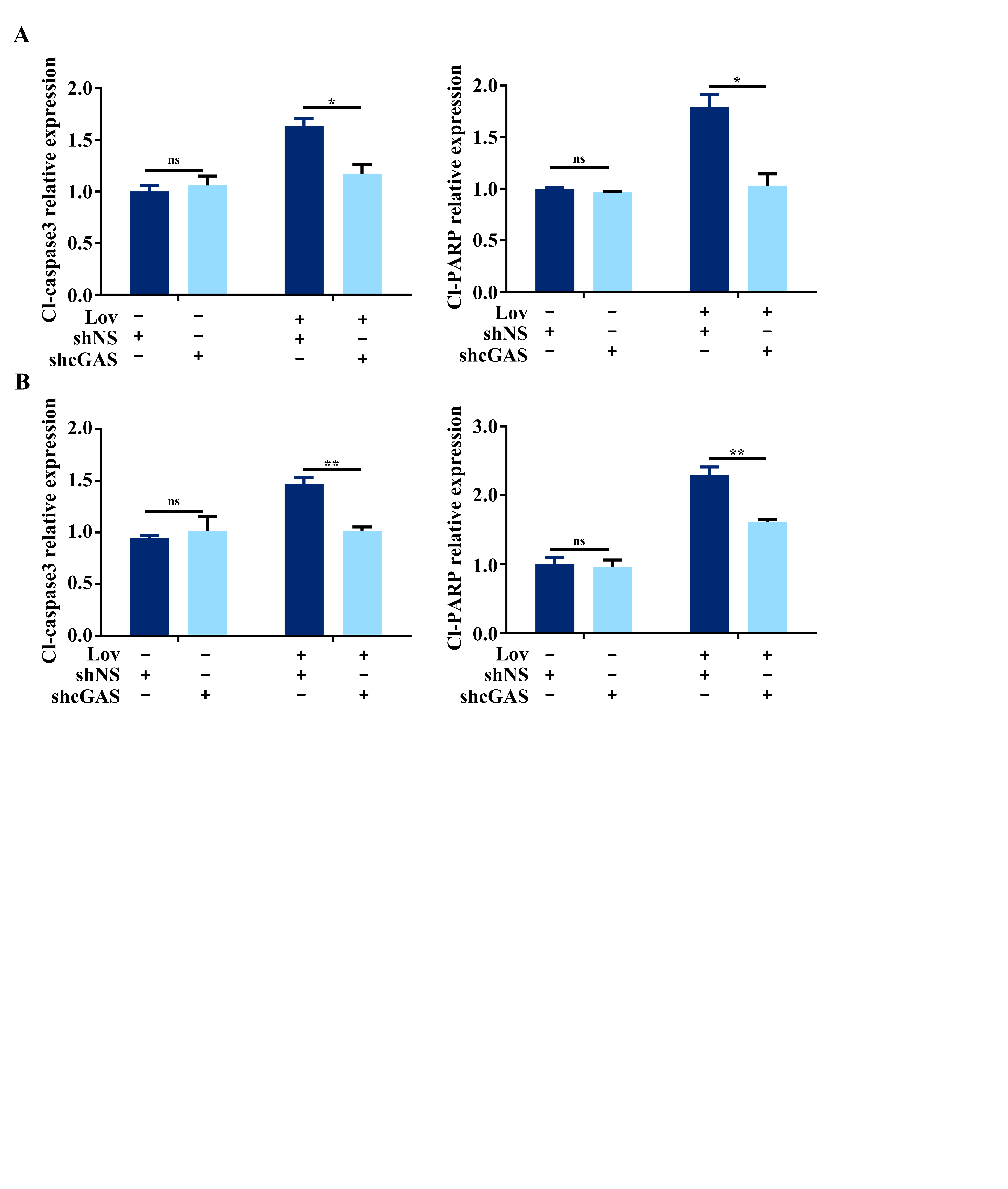

Supplement: Supplementary file 1 [file antioxidants-13-00679-s001.zip › Figure S5.tif]
